# Artificial intelligence in college students’ mental health education: opportunities, challenges, and strategic responses

**DOI:** 10.3389/fpsyg.2026.1794568

**Published:** 2026-05-11

**Authors:** Rui Wang, Xiaobo Sun, Yuqi Sun, Jing Wang

**Affiliations:** College of Chemical Engineering, Huaqiao University, Xiamen, China

**Keywords:** AI in higher education, college student mental health education, digital inclusion, ethical governance of AI, personalized mental health support

## Abstract

Mental health challenges among college students have become a growing global concern, placing increasing pressure on higher education systems to provide effective, scalable, and equitable mental health education and support. This narrative review examines the opportunities, challenges, and strategic responses associated with integrating artificial intelligence (AI) into college students’ mental health education. To improve conceptual clarity, it distinguishes educationally oriented AI applications from adjacent domains, including prevention-oriented support, early detection and screening, and supportive quasi-clinical intervention. The review identifies three main opportunity domains: personalized support for self-regulation and mental health learning, AI-assisted early awareness and prevention, and expanded accessibility and institutional reach. It also highlights key challenges related to ethical governance and privacy, accuracy and reliability, and digital divide and inclusivity issues. In response, the review emphasizes the need to strengthen ethical frameworks, improve system reliability and cultural responsiveness, and promote digital and AI literacy within higher education. Overall, current evidence suggests that AI can serve as a valuable complement to professional services and human-centered educational practices. However, the evidence base remains largely short-term and context-dependent, underscoring the need for longitudinal research, stronger institutional governance, and more inclusive implementation strategies. Responsible AI integration therefore requires a balanced, education-centered approach aligned with equity, student well-being, and institutional responsibility.

## 1 Introduction

Mental health problems among college students have become a major public health and educational concern worldwide. Large-scale epidemiological studies, including the World Health Organization’s World Mental Health International College Student surveys, show that roughly one-third of first-year college students meet criteria for at least one lifetime mental disorder, with similarly high rates of 12-month disorders, most commonly depression, anxiety, and substance use disorders (Auerbach et al., 2018; Bruffaerts et al., 2018). In the United States, diagnoses and help-seeking have continued to rise over the past decade, placing growing pressure on already stretched campus counseling systems (Oswalt et al., 2020; Lipson et al., 2022). These burdens are not evenly distributed. First-generation college students and students from marginalized racial and ethnic groups report higher levels of psychological distress and lower access to adequate support, pointing to persistent structural inequalities within higher education (Liu et al., 2019). Mental health difficulties during the college years are also linked to poorer academic functioning, lower persistence, and diminished well-being, making student mental health central to both educational success and institutional outcomes (Kalkbrenner et al., 2021). The digitalization of student life has introduced additional risks. Problematic internet use and internet addiction, for example, have been associated with emotional dysregulation and suicidal ideation among college students (Kang et al., 2023).

Alongside these developments, higher education is being reshaped by rapid advances in artificial intelligence (AI), which are altering teaching, learning, and institutional practice while opening new possibilities for mental health education in digitally mediated environments (Kamalov et al., 2023; Frank and Goodman, 2025). Existing review evidence suggests that AI in higher education may affect not only learning processes but also broader dimensions of student well-being, with potential benefits such as personalization and support coexisting with risks including technostress, digital fatigue, loneliness, and reduced face-to-face interaction (Klimova and Pikhart, 2025). Against this backdrop, the limitations of traditional mental health service models have become increasingly visible. Shortages of trained professionals, stigma surrounding help-seeking, and the difficulty of scaling face-to-face interventions have prompted growing interest in AI as a complement to existing support systems. Early work on AI-assisted online therapy and conversational agents such as Woebot and Wysa helped establish the feasibility and acceptability of low-threshold digital support formats (D’Alfonso et al., 2017; Fitzpatrick et al., 2017; Inkster et al., 2018). These studies, however, are better understood as early proof-of-concept or short-term support evidence than as direct evidence of sustained mental health education outcomes in higher education. A smaller body of work has also explored how AI may be incorporated into course design and broader educational settings, although such studies remain exploratory and context-specific rather than constituting strong evidence of educational effectiveness (Shan and Liu, 2021; Cai and Tang, 2022). More recent integrative work has therefore tended to frame AI not as a replacement for professional care, but as a supportive component of higher education mental health ecosystems, particularly in prevention-oriented education and early awareness (Zhai S. et al., 2025).

The integration of AI in college students’ mental health education can be examined through three interrelated dimensions: opportunities, challenges, and strategic responses. Here, “college students’ mental health education” refers to educationally oriented activities, resources, and institutional practices that strengthen students’ mental health literacy, emotional self-regulation, coping capacity, early awareness of distress, help-seeking readiness, and engagement with supportive resources. This definition is narrower than the broader domain of student mental health care. Accordingly, the review distinguishes among four related but non-identical domains: (1) mental health education in the strict educational sense; (2) prevention-oriented supports that promote resilience and early awareness; (3) screening and early detection tools that identify potential risk; and (4) quasi-clinical or supportive interventions, such as chatbot-based self-help systems. Studies from these adjacent domains are included only when they inform how AI may shape the broader educational ecosystem of student mental health, and they are not treated as interchangeable with direct evidence on educational outcomes.

To inform this analysis, relevant literature was identified through Web of Science, PubMed, Scopus, and Google Scholar, with primary emphasis on studies published between 2019 and 2026. Search strings combined terms related to AI, mental health, higher education populations, prevention, and digital literacy, and reference list screening was used to identify additional relevant sources. Because this article is a narrative critical review rather than a formal systematic review or meta-analysis, the aim was not exhaustive aggregation but transparent scope definition and comparative evidentiary interpretation. Studies were included when they focused on college or university student populations and examined AI-related approaches relevant to mental health education, prevention, early awareness, support, or implementation in higher education settings. Studies were excluded when they focused exclusively on non-student populations, unrelated educational technologies, purely technical algorithm development without clear educational or mental health relevance, or non-scholarly commentary. To improve analytical rigor, the synthesis differentiated among experimental and longitudinal studies, pilot and feasibility studies, observational studies, predictive modeling studies, and conceptual or review papers. In total, 93 sources were included in the final synthesis. The reporting of this narrative review was also informed by principles emphasized in SANRA, particularly transparency of scope, search description, and scientific reasoning (Baethge et al., 2019).

**Figure 1:**
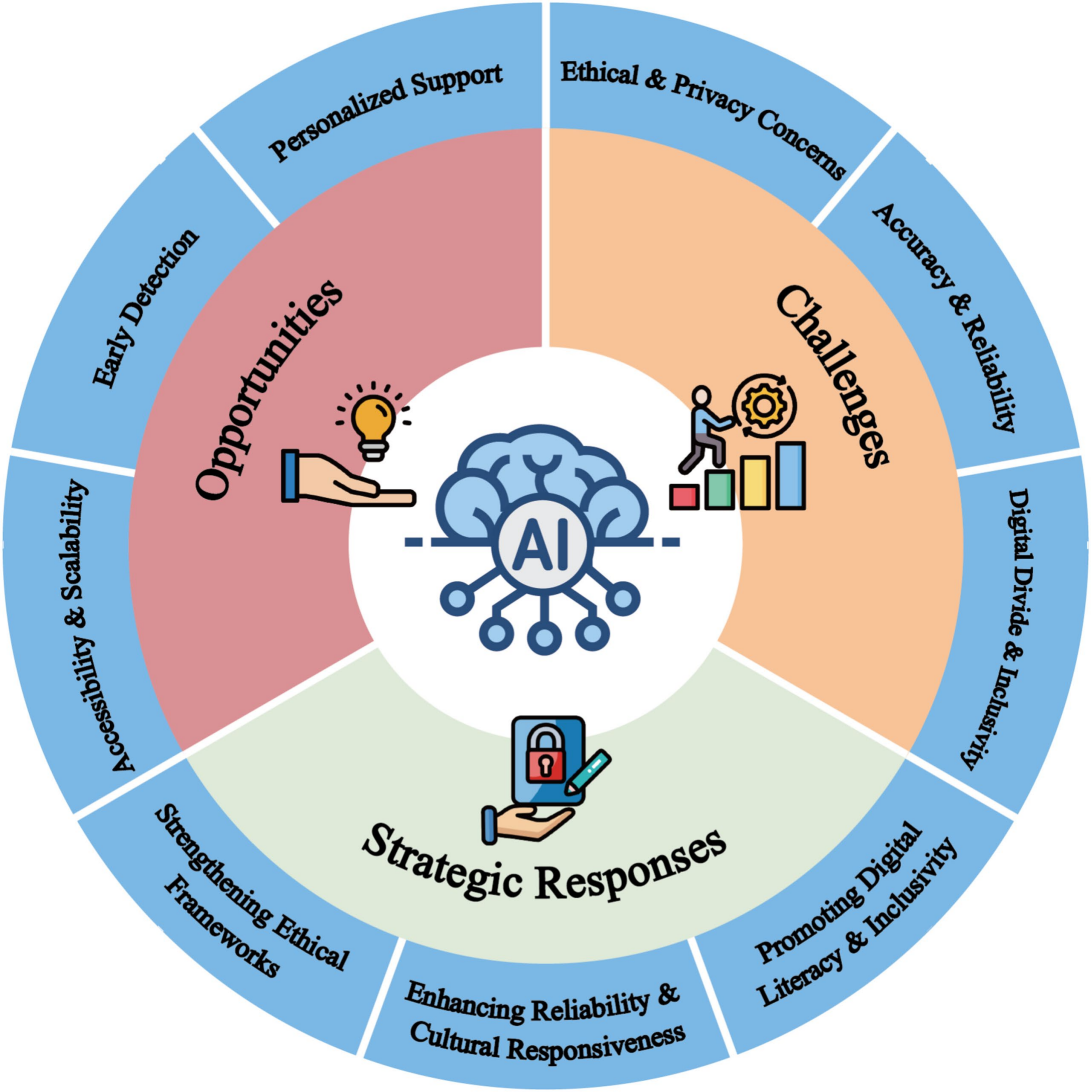
Conceptual framework of AI in college students’ mental health education. The diagram illustrates how AI applications provide opportunities, encounter challenges, and require strategic responses. Collectively, these dimensions highlight the transformative potential of AI in higher education mental health while underscoring the need for responsible, equitable, and human-centered implementation.

A mechanism-based educational psychology perspective further informs this analysis. Rather than assuming that AI tools directly improve student well-being or educational outcomes, this perspective treats AI as educationally relevant only when it creates need-supportive conditions that strengthen autonomy, competence, and relatedness. Within such conditions, AI-related functions such as personalization, adaptive feedback, reflective prompting, and low-threshold access may contribute to more proximal mechanisms, including emotional self-efficacy, mental health literacy, help-seeking readiness, and sustained engagement. These processes may in turn support preventive learning, coping behavior, and student well-being when embedded in human-supported higher education environments. The framework therefore serves as an interpretive model for organizing uneven evidence rather than as a fully confirmed causal theory. Consistent with this indirect interpretation, recent higher education evidence suggests that technical and teacher support contribute to AI literacy primarily through autonomy and competence need satisfaction rather than through direct effects alone (Shen and Cui, 2024). Throughout the review, conclusions about student experience, uptake, literacy, and educational relevance are anchored as far as possible in field-specific evidence drawn from higher education contexts. By contrast, evidence from adjacent domains such as healthcare AI, digital inequality research, and general AI ethics is used more cautiously to inform interpretation, likely risks, and governance-oriented recommendations rather than as direct proof of effect in college student populations. An overview of the main opportunities, challenges, and strategic responses discussed is presented in Figure 1.

## 2 Opportunities of AI in college students’ mental health education

The literature reviewed in this section is methodologically heterogeneous and should not be read as offering uniform levels of support. Randomized controlled trials provide the strongest direct evidence for short-term outcome claims, whereas pilot and feasibility studies are more informative about usability, acceptability, and engagement. Cross-sectional and correlational studies help identify associations and possible mechanism-related variables, but they do not establish causal educational effects. Predictive modeling studies primarily inform risk stratification and institutional decision support, while conceptual and review papers contribute interpretive, theoretical, and implementation-oriented perspectives rather than direct effect evidence. Accordingly, the synthesis below distinguishes among evidence for short-term symptom-related effects, user engagement and perceived usefulness, mechanism-related variables, and genuinely educational outcomes in higher education settings. The three opportunity domains discussed here are therefore not interchangeable. Some bear more directly on educational processes such as literacy, self-regulation, and participation, whereas others contribute more indirectly through prevention, early awareness, screening, or supportive intervention pathways.

### 2.1 Personalized mental health support

Among the opportunity domains most directly connected to mental health education, AI-driven tools may support personalized self-regulation, reflection, and low-threshold access to coping resources in higher education. Much of this literature, however, overlaps with supportive self-help intervention rather than education in the strict sense. The key distinction is therefore between educationally relevant functions, such as strengthening self-management and learning-oriented engagement, and quasi-clinical functions, such as symptom-focused chatbot assistance.

A substantial part of the evidence concerns conversational agents that adapt content through natural language processing and cognitive-behavioral principles while preserving a confidential and accessible format. The strongest direct evidence comes from short-term intervention studies. A 16-week randomized controlled trial found that a chatbot-delivered self-help intervention produced greater reductions in depressive and anxiety symptoms than bibliotherapy and was associated with a stronger perceived working alliance, although attrition remained moderate at about one quarter of participants (Liu et al., 2022). Pilot and feasibility studies point in a similar direction but are more limited in what they can support. For example, the Tess trial reported high usability and acceptability and substantial message exchange, yet symptom improvements were modest and dropout exceeded 50%, making it more informative about feasibility and participation constraints than about robust effectiveness (Klos et al., 2021). This pattern is consistent with recent review-level evidence. A rapid systematic review of nine studies involving 1,082 participants concluded that chatbot interventions for college students are promising but methodologically uneven, with substantial heterogeneity, high attrition, and a strong reliance on self-reported outcomes (Nyakhar and Wang, 2025). Meta-analytic evidence on digital mental health interventions in university populations likewise indicates beneficial short-term effects on depression and anxiety symptom severity, but this evidence still speaks more clearly to symptom-related outcomes than to educational effectiveness in higher education contexts (Madrid-Cagigal et al., 2025).

A different layer of evidence concerns perceived value and likely uptake rather than outcome effectiveness. Survey data suggest that many university students view chatbots and mental health apps as accessible, non-judgmental, and potentially useful complements or alternatives to face-to-face services, especially when they are reluctant to engage with traditional counseling (Gbollie et al., 2023). Personalization may also extend beyond symptom-focused tools to broader developmental and preventive formats. AI-enhanced life-crafting interventions, for instance, combine goal setting, value clarification, and personalized feedback in ways intended to strengthen purpose, self-regulation, and well-being without framing participation as therapy (Dekker et al., 2020).

Mechanism-oriented evidence adds another layer of interpretation. In a cross-sectional survey of 3,859 Chinese university students, engagement with AI-based mental health tools was positively associated with psychological well-being, with emotional self-efficacy and perceived autonomy in mental health management operating as parallel mediators (Sun et al., 2025). Because the study is cross-sectional, these findings are best treated as associational rather than causal. A similar logic appears in adjacent higher education AI literacy research, where motivational commitment was positively associated with AI literacy and self-efficacy served as a significant mediator (Kong et al., 2025). Although not specific to mental health education, this study reinforces the educational relevance of efficacy-based pathways when students engage with AI-supported learning environments.

Evidence on personalized AI-based support is promising but uneven. The strongest direct evidence concerns short-term symptom-related benefits in selected chatbot-supported self-help interventions, though these findings are limited by attrition and short follow-up. A second layer of evidence supports usability, acceptability, and perceived accessibility, suggesting that students may regard such tools as low-threshold and non-judgmental resources. A third layer concerns mechanism-related variables, including emotional self-efficacy, perceived autonomy, and self-regulation, but most of this work remains observational and cannot establish educational effectiveness by itself. Direct evidence for sustained educational outcomes in higher education remains limited.

### 2.2 Early detection and prevention

AI-based approaches to early detection and prevention occupy a related but analytically distinct position from mental health education in the narrow sense. They are included here not because screening and predictive analytics are themselves forms of education, but because they may shape the institutional conditions under which students develop early awareness of distress, encounter preventive resources, and engage with support pathways within higher education.

Recent student-focused modeling studies collectively suggest that AI can assist early risk stratification by integrating demographic, lifestyle, and behavioral indicators. Externally validated models trained on multi-institutional student samples have shown strong discriminative performance in predicting severe anxiety or depression (Zhang et al., 2024). Application-oriented work with incoming students similarly suggests that machine learning may help identify psychologically vulnerable freshmen during the transition into university life, although the generalizability of institution-specific models remains uncertain (Ma et al., 2026). Other studies have reported that multidimensional student data can be used to identify depressive symptom risk, reinforcing the promise of AI-assisted stratification while also highlighting the importance of careful feature selection and context-sensitive interpretation (Yu et al., 2025). This pattern is supported by broader review evidence. A systematic review focused on undergraduate populations found that machine learning is increasingly being used to detect anxiety, depression, and stress, but also noted substantial heterogeneity, variable data quality, and inconsistent validation practices (Schaab et al., 2024). These studies support the preventive relevance of AI-assisted early detection, while still falling short of direct evidence for long-term educational outcomes.

Beyond single-model demonstrations, machine learning-based prediction may also inform the allocation of preventive and educational resources at scale. Large national datasets have produced models with moderate but meaningful accuracy in identifying students at risk for diagnosable anxiety and depressive disorders based on socioeconomic and campus-related variables, supporting targeted outreach and early intervention by counseling and educational services (Zhai Y. et al., 2025). These models rely largely on routinely collected, nonclinical data, which makes them attractive in educational contexts where self-initiated help-seeking is limited. At the same time, explainable AI research suggests that predictive accuracy alone is not enough in student mental health settings; models must also be interpretable enough to support ethically defensible screening, communication, and intervention planning (Tariq et al., 2025).

AI-supported early detection also extends beyond predictive modeling to digital self-monitoring and reflective tools. A recent systematic review across multiple AI applications in mental health highlights the role of natural language processing-based journaling, behavioral tracking, and digital self-assessment tools in promoting early symptom awareness and self-reflection, especially among young adults (Dehbozorgi et al., 2025). These tools may encourage timely recognition of distress and engagement with educational or support resources, although the underlying evidence remains methodologically variable. Conceptual and applied work has also proposed integrating AI-supported early detection with behaviorally informed frameworks to guide low-intensity preventive interventions that strengthen coping skills and resilience before symptoms become severe (Zafar, 2024). Such work is useful for interpretation, but it should not be confused with direct empirical evidence of effect.

Collectively, AI appears capable of contributing to early awareness and prevention, but the nature of that contribution must be specified carefully. Predictive modeling studies primarily support risk stratification and institutional decision support rather than educational outcomes in the strict sense. Digital self-monitoring and reflective tools may be more directly relevant to preventive learning and self-awareness, but the evidence base remains heterogeneous and often limited in long-term follow-up. Conceptual work supports the plausibility of linking early identification with low-intensity preventive support, yet that plausibility is not equivalent to demonstrated effect. The field therefore supports the preventive relevance of AI-assisted early awareness, but not equally strong conclusions about long-term educational or mental health outcomes.

### 2.3 Accessibility and scalability

Accessibility and scalability are best understood as enabling conditions for mental health education and preventive support rather than educational outcomes in themselves. AI-supported digital interventions are relevant in this sense because they can broaden reach, reduce barriers to engagement, and extend the institutional availability of mental health learning and support resources. Mobile apps, web-based platforms, and text-based tools can reduce barriers related to cost, stigma, time constraints, and geographic distance, making them especially useful where timely support remains limited and shortages of mental health professionals persist (Lattie et al., 2022).

AI may further enhance scalability by enabling asynchronous delivery and adaptive support without continuous clinician involvement. Human-AI collaborative systems illustrate this potential. In a large non-clinical randomized trial, an AI-in-the-loop peer support system providing just-in-time feedback increased expressed empathy by 19.6%, suggesting that AI can strengthen human supportive capacity rather than replace interpersonal support (Sharma et al., 2023). Accessibility and scalability, however, do not by themselves ensure sustained engagement or meaningful benefit. Attrition remains a persistent problem in digital mental health interventions, and evidence on engagement is still limited by inconsistent metrics and weak links between engagement and outcomes (Smith et al., 2025). Broader synthesis work similarly suggests that the value of smartphone apps, generative AI systems, and other asynchronous tools depends heavily on implementation quality, integration with human support, and attention to equity and contextual fit (Torous et al., 2025). AI-supported tools are more effective when embedded in interactive pedagogies and scaffolded feedback structures rather than deployed as stand-alone technologies (Long et al., 2026).

Current evidence suggests that AI-enabled digital tools can improve reach and reduce barriers to access, which is an important enabling condition for mental health education and early support in higher education. Yet accessibility and scalability should not be conflated with effectiveness. Evidence for broad reach is stronger than evidence for sustained engagement, and evidence for sustained engagement is stronger than evidence for durable educational or well-being outcomes. The literature therefore supports the institutional value of AI for expanding access, but only more qualified claims about long-term impact.

The literature supports a differentiated rather than uniform interpretation of AI in college students’ mental health education. The strongest direct evidence concerns selected short-term symptom-related effects in chatbot-supported or digitally delivered self-help contexts. A broader but methodologically mixed body of research supports AI’s potential to improve accessibility, acceptability, and perceived usefulness. Evidence for mechanism-related variables, including autonomy, emotional self-efficacy, engagement, and mental health literacy, is promising but remains less mature and often observational. By contrast, direct evidence for sustained educational outcomes in higher education settings remains limited. The opportunities identified in this review should therefore be understood as unevenly supported across outcome domains rather than as equally established benefits.

Beyond access and scalability, the educational value of AI-supported tools can be understood more clearly through a mechanism-based educational psychology framework. From this perspective, self-determination theory provides a useful interpretive lens because it explains why students are more likely to engage meaningfully when educational environments support autonomy, competence, and relatedness. This view is consistent with broader educational evidence showing that self-determination-theory-based interventions can strengthen autonomous motivation and basic psychological needs (Wang et al., 2024), as well as with AI-specific higher education research showing that supportive university environments foster motivation to learn with AI and, in turn, stronger engagement in AI-assisted learning (Li et al., 2025).

**Figure 2:**
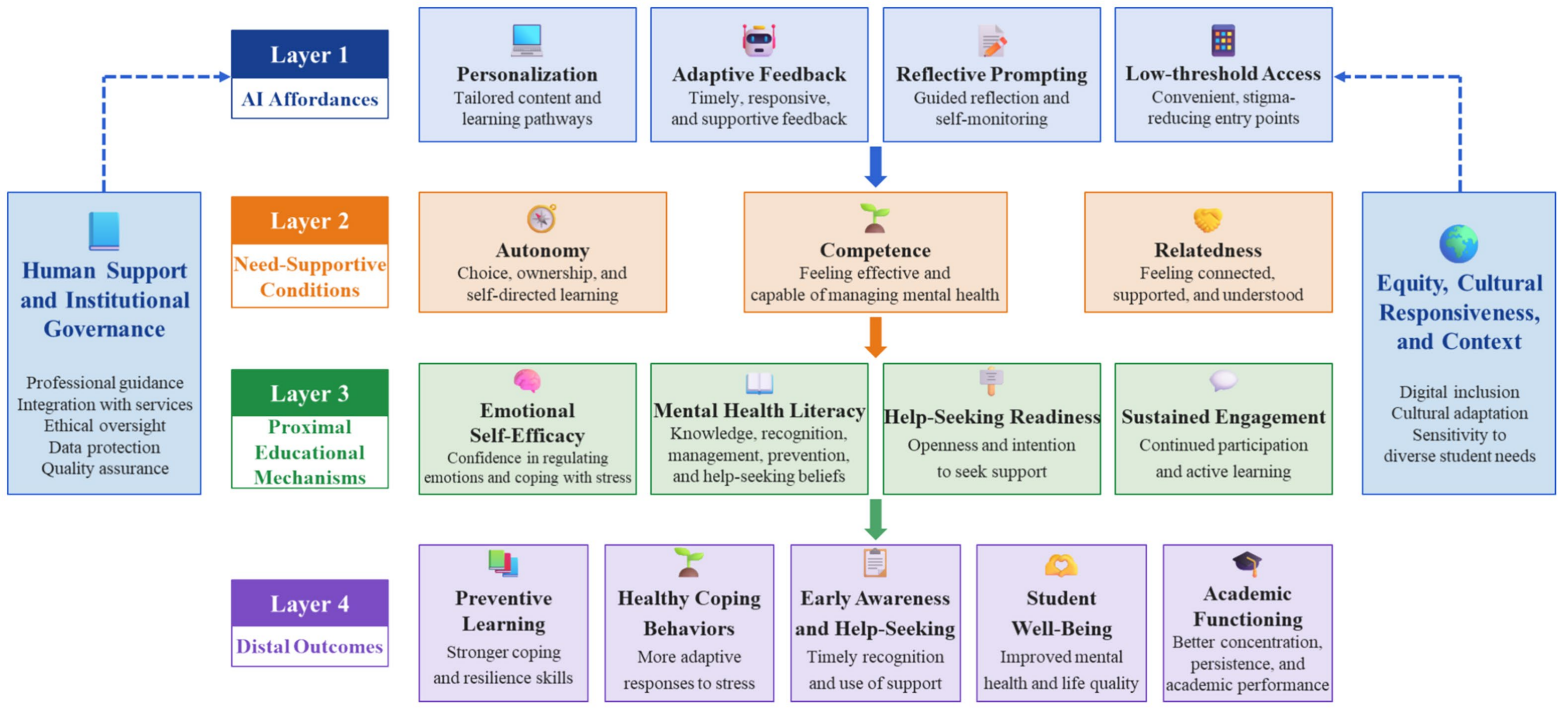
Mechanism-based educational psychology model of AI-supported mental health education for college students. The figure illustrates how AI affordances may contribute to educational and preventive value indirectly by supporting autonomy, competence, and relatedness, which in turn shape proximal mechanisms such as emotional self-efficacy, mental health literacy, help-seeking readiness, and engagement. The model also emphasizes that these pathways are conditioned by human support, institutional governance, and contextual inclusivity.

From this perspective, AI matters educationally not because it is merely personalized, scalable, or data-driven, but because these features may operate in need-supportive ways. In AI-supported mental health education, personalization and adaptive feedback may strengthen perceived autonomy and competence, while reflective tools and accessible support may contribute to mental health literacy, help-seeking readiness, and sustained engagement. Help-seeking readiness, in particular, should not be taken for granted. Cross-national evidence from 4,960 university students in Portugal, Germany, and Sweden shows that uncertainty about where to seek help is common and readiness to use internet-based interventions remains low (Pinho et al., 2025). As illustrated in Figure 2, these processes are best understood as plausible mediating mechanisms rather than guaranteed outcomes of AI use.

## 3 Challenges in integrating AI into mental health education

The challenge literature reviewed below includes both field-specific evidence from higher education mental health contexts and adjacent evidence from related domains such as healthcare AI, digital inequality research, and general AI ethics. These forms of evidence are not treated as equivalent. Field-specific studies are used to support claims about student experience, uptake, trust, and implementation in higher education, whereas adjacent literatures are used more cautiously to identify likely risks, interpretive concerns, and governance implications that may be relevant to college mental health education but do not, by themselves, demonstrate effects in student populations.

### 3.1 Ethical concerns and privacy

The use of AI in college students’ mental health education raises ethical concerns because it combines sensitive psychological information with institutional decision-making and unequal power relations between students, platforms, and universities. AI-supported systems may rely on extensive data collection, including behavioral traces and inferred emotional states, which can conflict with confidentiality, autonomy, and informed consent. In educational settings where trust is essential, opaque algorithmic processes and limited user control over data use may reduce students’ willingness to engage with AI-based mental health resources (Köbis and Mehner, 2021). Evidence from higher education also suggests that students and faculty already perceive data privacy, algorithmic bias, and unequal access to AI-supported resources as salient concerns, rather than merely abstract governance issues (Marín et al., 2025; Schmidt et al., 2025).

Privacy is especially important in mental health-related applications because unintended data exposure, secondary use, or weak safeguards may lead to stigma, mistrust, or psychological harm. Reviews of AI in mental health show that the same systems that enable personalization and early identification can also increase vulnerability to data misuse and inadequate anonymization, particularly outside regulated clinical environments (Poudel et al., 2025). For higher education, this makes privacy-by-design and data minimization central rather than optional features of responsible implementation.

Bias presents a related challenge. Models trained on imbalanced or culturally narrow datasets may misread emotional expression or produce systematically weaker performance for marginalized student groups. Research in AI and education shows that such problems are not simply technical errors but are shaped by broader social and institutional conditions, which means that fairness, accountability, and transparency must be treated as design and governance issues as well as modeling issues (Akgun and Greenhow, 2022).

Direct empirical evidence on AI ethics in college students’ mental health education remains limited. For that reason, adjacent work in AI ethics, moral psychology, and educational governance is most useful as an interpretive and governance-oriented resource rather than as direct evidence of student outcomes. International and education-focused frameworks converge on the importance of human oversight, participatory governance, and student recognition as stakeholders (Nguyen et al., 2023). Work in moral psychology also suggests that generative AI may encourage misplaced trust or moral delegation, which is particularly relevant in mental health education settings where users may be vulnerable and support-related decisions carry emotional weight (Bonnefon et al., 2024; Salah et al., 2024). The ethical challenge is not only to prevent harm, but also to ensure that AI-supported mental health education remains transparent, accountable, and worthy of student trust.

### 3.2 Accuracy and reliability of AI systems

Although AI-based tools promise scalable and real-time support for college mental health education, their accuracy and reliability remain a central concern. Performance depends not only on model design, but also on the quality, diversity, and contextual relevance of the data on which systems are built and evaluated. Recent review evidence from education reinforces this point: ethical concerns surrounding AI in education repeatedly converge on questions of bias, privacy, transparency, and weak empirical validation, indicating that reliability in educational settings cannot be treated as a purely technical matter (Yan et al., 2025). Reviews from mental health contexts point in a similar direction. Research on digital phenotyping, for example, shows that behavioral signals collected through smartphones and connected devices are inherently dynamic, incomplete, and context-sensitive, which makes stable interpretation difficult without robust analytical and validation frameworks (Torous et al., 2017). Reliability in AI-supported mental health education should therefore be understood as an ongoing problem of evaluation, context, and governance rather than as a one-time benchmark of model performance.

A further difficulty is that apparent predictive accuracy may mask uneven performance across student groups. Evidence from heterogeneous populations suggests that indicators of depression or distress do not operate uniformly across demographic and socioeconomic subgroups, leading to systematic variation in model performance and weaker reliability for some groups than for others (Adler et al., 2024). Adjacent work in medical AI extends this warning by showing that bias may accumulate across the entire AI lifecycle, including feature construction, labeling, model development, evaluation, deployment, and publication practices (Cross et al., 2024). These studies suggest that subgroup-aware validation is essential in higher education applications and that apparently strong overall accuracy should not be treated as sufficient evidence of fairness or robustness.

Reliability challenges are also visible in institutionally deployed systems. Applications of AI to large educational datasets show that rapid, large-scale mental health assessment is technically feasible, but also that outputs remain sensitive to data quality, model configuration, and calibration choices (Wang, 2023). In practice, this means that systems may appear precise while still varying in validity across cohorts or institutions. More broadly, review evidence from applied AI and machine learning emphasizes that trustworthy performance cannot be reduced to isolated benchmark scores; it depends on evaluation procedures that extend from development to deployment and include human interaction with the system in actual use settings (Hanna et al., 2025). For higher education, this makes transparent reporting, continuous recalibration, and human oversight central to responsible implementation.

Additional limitations arise when AI systems rely on proxy signals from online behavior and social media. A critical review of predictive techniques for mental health inference from social media highlights recurring problems of construct validity, inconsistent labeling, and weak ground-truth verification, all of which reduce confidence in model outputs (Chancellor and De Choudhury, 2020). User experience introduces another layer of uncertainty. Qualitative evidence suggests that students are sensitive to repetitive, generic, or contextually inappropriate AI responses, which can quickly erode trust in AI-supported mental health resources (Chan, 2025). The available literature suggests that accuracy and reliability in AI-supported mental health education are best treated as context-sensitive and continuously negotiated rather than as fixed technical properties. Direct higher education evidence is growing, but it remains narrower than the broader literature on digital mental health prediction and AI reliability.

### 3.3 Digital divide and inclusivity issues

Despite the rapid expansion of AI-enabled mental health tools, their benefits are unlikely to be distributed evenly across student populations. Review evidence on digital mental health interventions for college students shows that, although many programs appear promising, uptake, engagement, and implementation remain uneven, and there is still limited evidence that benefits are equitably realized at scale (Lattie et al., 2019). In this context, availability alone does not ensure inclusion. What matters is whether students can access, understand, trust, and meaningfully use AI-supported mental health resources.

These inequalities increasingly take AI-specific forms. Recent higher education research suggests that students differ not only in access to AI tools, but also in their knowledge, confidence, and ability to turn AI use into educational advantage. Studies of generative AI use identify distinct student profiles, ranging from novice and cautious users to more confident and strategic users, indicating a new layer of digital inequality within higher education (Beckman et al., 2025). Cross-national evidence similarly shows that most university students possess only foundational AI literacy, with meaningful differences across countries in AI literacy, AI self-efficacy, and attitudes toward AI (Hornberger et al., 2025). These findings suggest that AI-supported mental health education may reproduce inequality unless institutions address variation in students’ AI-related capabilities and readiness.

Broader forms of digital and health-related literacy also shape participation. First-generation college students report lower digital proficiency than their peers, and digital proficiency is positively associated with psychological well-being (Deng and Yang, 2021). Related evidence shows that stronger digital literacy is linked to better mental well-being and lower self-isolation in university populations (Tek and Özsari, 2025). In health-related settings, students with higher eHealth literacy are better able to evaluate digital health information and tend to view AI tools as more useful, even while expressing concern about risks and over-reliance (Alam et al., 2025). Together, these findings suggest that AI-supported mental health education depends partly on whether students possess the digital, informational, and evaluative skills needed to engage with such tools critically.

Inclusivity challenges also appear in actual uptake. Among first-year college students, attitudes toward mobile mental health interventions are often more positive than real-world use, and use tends to be higher among those already connected to formal mental health services (McCarthy and Horwitz, 2025). Reported barriers include concerns about credibility, usability, customization, and the lack of human guidance. This gap matters because it suggests that students who are already closer to institutional support may benefit most, while those with lower problem recognition or weaker support pathways may remain underserved.

Adjacent work on AI and the digital divide in education further suggests that exclusion may persist even when nominal access exists, because language barriers, cultural mismatch, and algorithmic bias can reduce usability and trust for marginalized groups (Matjie et al., 2026). A related concern appears in discussions of AI in mental healthcare, where accessibility is seen as valuable only when relational support is preserved rather than displaced (Babu and Joseph, 2024). For college mental health education, the implication is clear: inclusive implementation requires more than expanding access. It also requires literacy-building, culturally responsive design, and continued human support so that AI functions as a complement to, rather than a substitute for, supportive educational environments.

The digital divide in AI-supported mental health education is not simply a matter of access. It also reflects unequal levels of literacy, confidence, trust, and support, all of which shape whether students can benefit from AI-enabled resources. Addressing these barriers requires literacy-building, culturally responsive design, and continued human support. Table 1 summarizes these challenges, their likely consequences, and the corresponding strategic responses discussed in the next section.

**Table 1:** Key challenges in integrating AI into college students’ mental health education and corresponding strategic responses.

Challenge	Specific issues	Potential consequences	Strategic responses
Ethical concerns and privacy	Data ownership, lack of transparency, algorithmic bias, risk of misuse	Loss of trust, privacy breaches, reinforcement of inequities	Strengthening ethical frameworks: transparency, fairness, ethics of care, clear accountability
Accuracy and reliability	Limited representativeness of training data, digital phenotyping noise, proxy signals from social media	Misclassification of mental states, unreliable predictions, reduced validity across diverse groups	Enhancing AI reliability: rigorous validation, bias mitigation, interdisciplinary collaboration, continuous monitoring
Digital divide and inclusivity	Gaps in access, digital literacy, usability, and cultural responsiveness	Exclusion of disadvantaged groups, exacerbation of mental health inequalities	Promoting digital literacy and inclusivity: equitable access, skill-building programs, participatory design, culturally responsive systems

The challenges and responses presented in this table are summed up from the literature reviewed in Sections 3 and 4; detailed references are provided in the main text.

## 4 Strategic responses and recommendations

**Figure 3:**
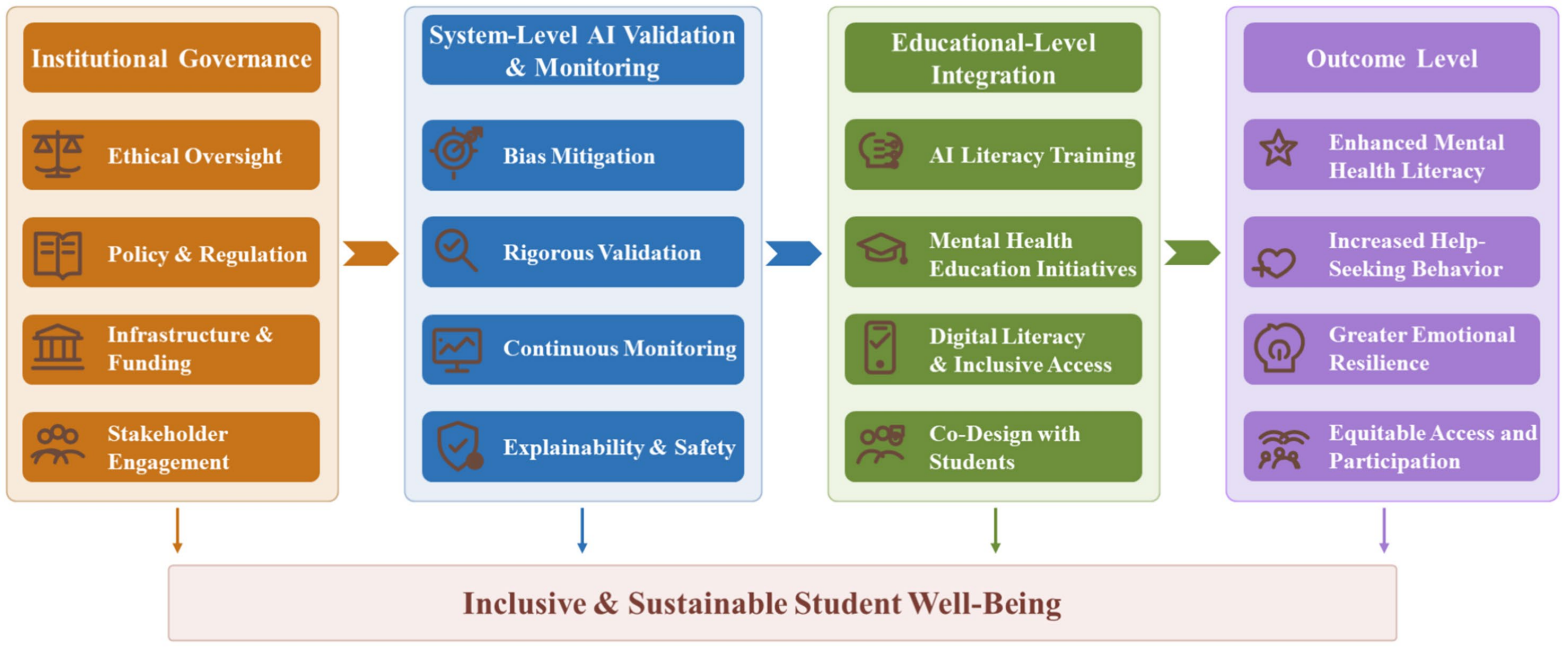
Institutional implementation pathway for integrating AI into college students’ mental health education. The figure illustrates how institutional governance, system-level validation and monitoring, and educational-level integration interact to support inclusive and sustainable student well-being outcomes.

The responsible integration of AI into college students’ mental health education requires coordinated action at multiple levels. Because field-specific intervention evidence remains limited in several areas, these recommendations draw on both higher education research and cautiously interpreted insights from adjacent fields, particularly healthcare AI, AI ethics, and digital inequality research. They are therefore intended as proportionate guidance for implementation rather than as claims that every recommendation has already been directly validated in college mental health education settings. As shown in Figure 3, this process involves institutional governance and infrastructure, system-level design and ongoing validation, and educational efforts to strengthen digital and AI literacy, with the broader aim of supporting inclusive and sustainable student well-being.

### 4.1 Strengthening ethical frameworks

Strengthening ethical frameworks is a basic requirement for the responsible use of AI in college students’ mental health education. International discussions of AI ethics increasingly converge around principles such as transparency, fairness, accountability, non-maleficence, and privacy. Yet broad agreement at the level of principle does not automatically translate into workable practice. This gap is particularly important in mental health-related educational settings, where trust, vulnerability, and autonomy are central. Existing research in both AI ethics and higher education shows a recurring set of concerns, including privacy and data protection, algorithmic fairness and bias, transparency and explainability, student well-being, and accountability through human oversight, while also noting the continuing lack of clear operational guidance across educational contexts (Jobin et al., 2019; Alfiras et al., 2025). Together, these findings suggest that higher education institutions need ethical frameworks that are not only principled, but also practical and context-sensitive.

For universities, this means that ethical governance cannot be reduced to formal policy statements. It requires clear decisions about how student data are collected and used, how algorithmic outputs are interpreted, and who is responsible for oversight. Research on higher education governance suggests that university responses to generative AI have generally been cautious, especially in relation to ethics, privacy, and system limitations, and that stronger knowledge-based institutional strategies are associated with clearer governance practices and better student legal and ethical awareness (Oncioiu and Bularca, 2025; Tong et al., 2025). In this sense, governance is not simply a compliance mechanism. It also shapes how students understand AI-related norms, risks, and responsibilities. In the context of mental health education, ethical frameworks should therefore be embedded in institutional cultures of transparency, shared understanding, and accountable oversight.

A further issue is that ethical principles must be built into the full lifecycle of AI systems rather than added after deployment. Work on AI ethics tools, student perceptions, and educational technology adoption points in the same direction: effective governance depends on integrating transparency, explainability, and human oversight into design, implementation, and ongoing monitoring, while also providing clear guidelines, ethics training, and safeguards against misuse (Morley et al., 2020; Barus et al., 2025; Nazaretsky et al., 2025). These measures matter not only for risk reduction, but also for cultivating trust, intelligibility, and responsible participation among students. From this perspective, the value of AI should be judged not only by technical performance but also by its fit with educational aims such as inclusion, student well-being, and empowerment (Floridi et al., 2020).

At the same time, principle-based ethics alone may be insufficient in contexts that involve emotional vulnerability and relational support. An ethics of care perspective draws attention to how AI systems may shape users’ emotional experiences, perceived support, and expectations of responsibility, especially when conversational systems simulate interpersonal interaction (Tavory, 2024). This is particularly relevant to mental health education, where the quality of support cannot be separated from questions of trust, dependency, and human presence. Strengthening ethical frameworks therefore requires more than a list of principles. It calls for a layered approach that combines shared norms, practical governance, and care-sensitive judgment so that AI remains a supportive and trustworthy complement to existing educational resources.

### 4.2 Enhancing AI systems reliability and cultural responsiveness

Improving the reliability of AI systems is essential if they are to play a responsible role in college students’ mental health education. Reviews of conversational agents in mental health have repeatedly shown wide variation in study design, outcome measures, and reporting practices, making it difficult to judge performance consistently or to generalize findings across settings (Vaidyam et al., 2019). Similar concerns appear in more recent reviews of large language models in mental health care, where non-standardized evaluation, limited comparability across studies, and the use of proprietary systems all make performance claims harder to interpret with confidence (Hua et al., 2025). These limitations suggest that reliability should not be understood simply in terms of reported accuracy, but in terms of whether systems are evaluated transparently, consistently, and in ways that fit their actual context of use. For higher education, this points to the need for clearer validation standards, transparent subgroup reporting, and continued refinement based on real-world use rather than pilot findings alone.

Questions of reliability are closely connected to questions of cultural responsiveness, especially in language-based systems. Research has shown that natural language processing tools used in mental health contexts can reproduce systematic biases related to race, gender, religion, nationality, and age, reflecting both skewed training data and culturally narrow assumptions about how distress is expressed (Straw and Callison-Burch, 2020). This concern extends to LLM-based mental health applications, where general-purpose models may generate inaccurate, biased, or stigmatizing responses unless they are adapted and assessed in ways that are sensitive to context and equity (Lawrence et al., 2024). In culturally and linguistically diverse higher education settings, such problems may distort students’ experiences and reduce the relevance and trustworthiness of AI-supported resources.

Addressing these problems requires more than technical adjustment at a single stage of development. Psychological science has emphasized the importance of routinely assessing bias, examining model performance across sociodemographic groups, and adopting mitigation strategies throughout model design, evaluation, and use (Timmons et al., 2022). Related review evidence from healthcare AI similarly highlights the importance of representative data, transparent decision processes, and interdisciplinary oversight in reducing bias and improving fairness (Chinta et al., 2025). In the present context, this means that psychologists, educators, data scientists, and ethicists need to work together so that system design is informed not only by technical performance, but also by student diversity and educational purpose.

Reliability also cannot be treated as something established once and for all before implementation. Review evidence from healthcare AI shows that bias may emerge over time through concept drift, changing user populations, or shifts in deployment context, which is why continued monitoring, external review, and periodic updating remain important after rollout (Hasanzadeh et al., 2025). A similar point is made in recent work on mental health LLMs, which argues that reliability depends on representativeness, interpretability, cultural sensitivity, and the support structures surrounding end users, not on model performance alone (Malgaroli et al., 2025). Although much of this literature comes from clinical settings, the underlying lesson is highly relevant to mental health education in higher education. Systems are more likely to be trusted and more likely to be useful when institutions treat reliability as an ongoing evaluative process and cultural responsiveness as a basic condition of responsible use.

### 4.3 Promoting digital literacy and inclusivity

Promoting digital literacy and inclusivity is a necessary condition for the responsible and equitable use of AI in college students’ mental health education. Inclusion cannot be reduced to nominal access to the internet or to AI tools. Research on digital inequality shows that access is also shaped by the quality and diversity of devices, the affordability of maintaining them, and the social support needed for effective use. In higher education, simply making AI tools available is therefore unlikely to reduce inequality if students differ substantially in the material and social conditions that shape their ability to benefit from those tools (van Deursen and van Dijk, 2018). More broadly, recent review evidence on AI in higher education suggests that equity, inclusivity, and digital fluency should be treated as central conditions of responsible implementation rather than as peripheral concerns (Figueroa de la Fuente and Farhadian, 2025).

A practical implication is that universities should treat digital literacy as a multidimensional educational capacity rather than a basic technical skill. Digital competence also includes information management, communication, collaboration, critical thinking, creativity, and problem-solving. These skills do not develop evenly and are shaped not only by background characteristics but also by training, self-directed learning, perceived ease of use, and support from others (van Laar et al., 2019). In mental health education, such capacities matter because students must be able to search for information, judge its credibility, communicate about distress, and engage with digital resources reflectively.

AI literacy is a related but distinct layer of preparedness. Within higher education, AI use, attitudes, and AI literacy are unevenly distributed, and both students and staff report a need for clearer institutional guidance and support for responsible use (Brown et al., 2025). Review evidence further suggests that AI literacy is associated with stronger self-efficacy, more positive attitudes toward AI, higher motivation, and better digital competencies, whereas lower AI literacy is associated with greater AI anxiety (Bewersdorff et al., 2025). This pattern is echoed in student evidence showing that engagement can strengthen AI literacy indirectly through reduced anxiety and enhanced self-efficacy (Farmanesh et al., 2025). In this context, AI literacy is not simply technical proficiency, but a basis for informed and appropriately bounded engagement with AI.

For mental health education more specifically, digital health literacy and digital mental health literacy also require explicit attention. Cross-national evidence indicates that many university students can navigate digital platforms at an operational level but are less prepared to evaluate the reliability of health information critically. Trust in information sources appears to matter more than mere exposure to online content (Ishikawa et al., 2025). Related research further suggests that digital mental health literacy and critical thinking help translate institutional support into more proactive mental health behaviors, including more informed help-seeking and self-management (Younis, 2025). Students benefit from digital tools when they can judge credibility, interpret outputs cautiously, and connect digital information with appropriate action.

Mental health literacy remains equally important within this broader framework. A recent systematic review shows that university students’ mental health literacy varies considerably and is shaped by stigma, cultural diversity, sociodemographic factors, psychiatric history, and prior experiences of psychological distress (Líbano et al., 2024). This suggests that literacy-building interventions should not assume a uniform student population. More inclusive implementation would combine broad literacy support with targeted adaptation for groups that face greater barriers to recognition, trust, or help-seeking.

An effective response requires a layered institutional approach. Universities should move beyond simply providing AI tools and instead strengthen students’ digital, AI, and mental health-related literacies through curricula, orientation, and support services. They should also provide clear guidance on appropriate AI use and improve access to trustworthy and culturally responsive mental health information. Inclusivity therefore depends not only on access, but also on whether students have the skills and support needed to benefit from AI fairly.

## 5 Future outlook and conclusion

AI is creating new possibilities for college students’ mental health education, especially in personalization, early awareness, and wider access to preventive support. At the same time, these possibilities are constrained by ethical, methodological, and social challenges, including privacy risks, uneven reliability, limited cultural responsiveness, and digital inequality. Progress in this field will depend not simply on technological innovation, but on whether AI is embedded in responsible, education-centered, and institutionally supported practice.

Several priorities follow for future research and practice. More longitudinal studies are needed in real higher education settings, particularly studies that move beyond short-term and self-reported outcomes to examine sustained educational, behavioral, and well-being effects. It is also important to distinguish more clearly between evidence on mental health education itself and evidence on prevention, early awareness, screening, and supportive intervention, since these domains are related but not interchangeable. In the current literature, the evidence is stronger for short-term support effects and mechanism-related processes than for durable educational outcomes in higher education.

The central theoretical argument is not that AI directly improves college students’ mental health education. Instead, AI is most likely to matter educationally when it supports autonomy, competence, and relatedness, thereby strengthening mental health literacy, emotional self-efficacy, help-seeking readiness, and sustained engagement. This perspective helps distinguish direct educational claims from adjacent claims drawn from prevention-, screening-, or support-oriented applications, and it clarifies why the present evidence base should still be interpreted with caution.

For higher education institutions, this means that AI should be treated as a complement to professional services and supportive educational environments, not as a substitute for them. Stronger claims should remain grounded in evidence from higher education contexts, while adjacent literatures are more appropriately used to inform interpretation, risk awareness, and implementation. Ethical oversight, human judgment, participatory governance, and efforts to strengthen digital, AI, and mental health literacy should therefore remain central to implementation. Under these conditions, AI may make a meaningful contribution to college students’ mental health education, but that contribution will depend on clear conceptual boundaries, responsible governance, and inclusive practice.
